# Graphene as a Transparent Conductive Electrode in GaN-Based LEDs

**DOI:** 10.3390/ma15062203

**Published:** 2022-03-16

**Authors:** Hehe Zhang, Jan Mischke, Wolfgang Mertin, Gerd Bacher

**Affiliations:** Werkstoffe der Elektrotechnik and CENIDE, Universität Duisburg-Essen, 47057 Duisburg, Germany; jan.mischke@uni-due.de (J.M.); wolfgang.mertin@uni-due.de (W.M.)

**Keywords:** graphene, GaN, LED, CVD, contact resistance, transparency, current spreading

## Abstract

Graphene combines high conductivity (sheet resistance down to a few hundred Ω/sq and even less) with high transparency (>90%) and thus exhibits a huge application potential as a transparent conductive electrode in gallium nitride (GaN)-based light-emitting diodes (LEDs), being an economical alternative to common indium-based solutions. Here, we present an overview of the state-of-the-art graphene-based transparent conductive electrodes in GaN-based LEDs. The focus is placed on the manufacturing progress and the resulting properties of the fabricated devices. Transferred as well as directly grown graphene layers are considered. We discuss the impact of graphene-based transparent conductive electrodes on current spreading and contact resistance, and reveal future challenges and perspectives on the use of graphene in GaN-based LEDs.

## 1. Introduction

GaN-based light-emitting diodes (LEDs) have been intensively studied for applications in the visible and ultraviolet (UV) spectral range [1,2,3,4]. GaN-based visible light LEDs can be used for general illumination, traffic signals, automobile headlights, and backlight units for liquid crystal displays [5], while GaN-based UV LEDs could be applied in germicidal instrumentation, biological agent identification, chemical sensing, fluorescence excitation, water disinfection and optical data storage [6,7].

Since the commonly used p-type cladding layers in GaN-based LEDs, such as Mg-doped GaN or AlGaN, have deep acceptors with an activation energy of, e.g., around 170 meV in GaN [8] or around 400 meV in Al_0_._7_Ga_0_._3_N [9], the p-cladding layer is not sufficiently conductive [2,10]. As a consequence, current crowding near the p-electrode edges occurs, which could severely deteriorate the stability and reliability of the LED devices [11,12,13,14]. For a more-homogeneous distribution of current, a transparent current spreading layer (TCSL) with low electrical sheet resistance and high optical transparency is essential [15]. In the early stage of GaN-based LED development, the commonly used p-contact material was Ni/Au. To overcome the current crowing problem, scientists attempted to directly use Ni/Au as a transparent conductive electrode and improved the transparency of thin Ni/Au films from around 50% to over 83% in the 400–750 nm spectra region by rapid thermal annealing (RTA) [16]. In the following, studies started to use transparent conductive oxides, such as indium tin oxide (ITO), as TCSL. ITO has both high conductivity and a high transparency of over 85% in the range of 400–600 nm [17,18]. However, as a commercial TCSL the cost increase of ITO due to the low abundance of indium on Earth (around 0.05 ppm) and its low recycling rate (below 10%) is detrimental [19]. Moreover, the intrinsic transmission loss of ITO below 400 nm [20,21] restricted its application in UV-LEDs, and the low thermal conductivity of 11~12 W/m·K of ITO [22] could lead to a high local temperature, potentially degrading the performance of high power GaN-LEDs [23]. Additionally, due to its mechanical brittleness ITO is not suitable for the use in flexible devices [24].

Graphene, defined as a two-dimensional (2D) carbon material consisting of a hexagonal array of sp^2^-hybridized carbon atoms with a single-atom-thickness, can exhibit a high transparency of >91% in a wide range from 200 nm to 1000 nm [25], a low sheet resistance ($R_{S}$) of ~250 Ω/sq [25] and a high thermal conductivity of ~2000–4000 W/m·K [26,27,28] that is several hundred times higher than that of ITO. Low-cost fabrication approaches [25,29,30] has made graphene already commercially available [31,32]. Based on these advantages, graphene is considered as a promising candidate for replacing ITO as TCSL in GaN-based LEDs, especially in the UV range. However, the up-to-date contact resistance between graphene and p-GaN is in the order of Ωcm^2^ [33,34,35], which could originate from the large work function mismatch between graphene of 4.2–4.5 eV [36,37,38] and p-type doped GaN (p-GaN) of ~7.5 eV [39]. This might inhibit the hole injection efficiency and could result in a higher operating voltage.

In the second decade of the 21st century, GaN-based LEDs with graphene as TCSL have been intensively studied. Different aspects of this research are reviewed in this work. The main efforts of these studies focused on the reduction of the contact resistance, and in keeping the low sheet resistance and high transparency of graphene-based TCSL after integration into GaN-based LEDs. In this review, the numerous efforts for integration of graphene-based TCSL in GaN-based LEDs with single- (SQW) or multi-quantum wells (MQW) are summarized. In Section 2, the improvement of properties of graphene-based TCSLs through varying the fabrication methods (Section 2.1) or by realizing graphene hybrid structures (Section 2.2) are described. The fabrication of graphene films includes both transfer (Section 2.1.1) and direct growth (Section 2.1.2) on GaN-based LEDs. Hybrid graphene structure consisting of graphene networked with ITO nanodots (Section 2.2.1), with metal interlayer, nanoparticles, nanowires (Section 2.2.2) and other graphene configurations (Section 2.2.3) are introduced. The operation mechanisms of the current spreading are discussed in Section 2.3. Section 3 presents the efforts on contact engineering for lowering the contact resistance, one of the main obstacles for integration of graphene-based TCSL in GaN-based LEDs. After a brief motivation (Section 3.1), the two main methods for reducing the interface barrier between graphene and p-GaN are illustrated, namely p-doping of graphene for work function adaptation (Section 3.2) and inserting a thin dielectric film (Section 3.3). Finally, advanced device structures of GaN-based LEDs with 3D architecture and graphene as TCSL are presented to provide a wider scope of this review.

## 2. Graphene as TCSL in GaN-Based LEDs

In this section we will present the efforts of integrating graphene either alone or in a hybrid structure serving as TCSL in GaN-based LEDs.

### 2.1. Enhancement of LED Performance by Graphene Film Integration

Here, the performance of a TCSL of graphene fabricated by either transfer or direct growth process is discussed. The fabrication details of transferred and transfer-free graphene films are reviewed in other works [40,41,42] and will not be repeated here.

#### 2.1.1. Transferred Graphene Films

**Figure 1:**
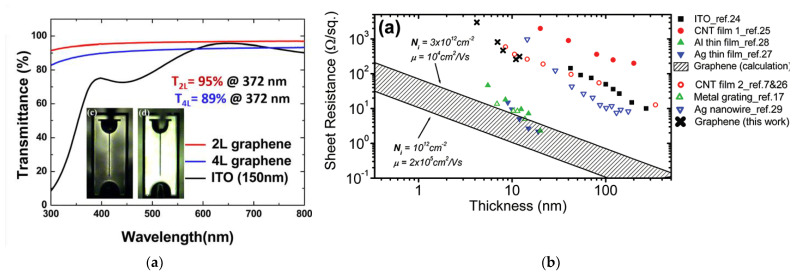
(**a**) Transferred graphene as TCSL integrated into GaN-based LEDs: Transmittance from 300 nm to 800 nm of 2L and 4L graphene in comparison to the typical transparent electrode ITO. Inset: optical images of light emission of a typical LED device (left) without and (right) with few-layer graphene. (**b**) Sheet resistance of different transparent conductors versus film thickness. (**a**) Reproduced with permission from [23]. Copyright 2011 American Institute of Physics. (**b**) Reproduced with permission from [48]. Copyright 2010 American Chemical Society.

As previously mentioned, single layer graphene (SLG) has a high theoretical (~97%) and experimental (≥91%) optical transmittance (T) over a wide spectral range from 200 nm to 1000 nm [25,43]. Even for two-layer graphene (2LG) and four-layer graphene (4LG) the transparency is easily over 80% from the ultraviolet to the infrared region, as shown in Figure 1a. In contrast, a dramatic transparency drop of a conventional ITO TCSL is observed below a wavelength of 400 nm. Transferred graphene films supply a good current spreading in GaN-based LEDs with respect to the bare GaN-based LEDs. An enlarged luminescence area around the p-contact electrode, as illustrated in the inset of Figure 1a, can be repeatedly observed in numerous works [23,44,45,46]. From a microscopic point of view, this improved distribution of injected holes through graphene in GaN-based MQW LEDs ensures a shorter transport time of holes along the lateral direction, a reduced capture time of holes by the quantum wells, which is revealed by a time-resolved electroluminescence (TREL) measurement [47].

Table 1 summarizes additional performance data of GaN-based LEDs with graphene TCSL, such as forward voltage ($V_{f}$), light output power (LOP) and electroluminescence (EL) intensity. The LOP in graphene-enhanced GaN-based blue LEDs is often not noticeably improved compared to ITO-GaN LEDs. From 2010 to 2021, only two reports verified graphene as a better TCSL compared to ITO regarding the LOP in blue (460 nm) [49] and UV (380 nm) [50] emitting LEDs. Most works revealed that the typical forward voltage of GaN-based LEDs with graphene as TCSL is larger than 5 V, whereas the value of the same LEDs with 250 nm ITO as TCSL ($V_{f,250\mathrm{nm}\;\mathrm{ITO}}$) is only, for example, 3.4 V [49]. Many studies showed either a lower LOP in the LEDs with graphene-only TCSL than with conventional ITO [51] or with thin Ni/Au TCSLs [33] or a lower EL intensity [15,52] (see Table 1).

To understand similarities and differences in the performance of GaN-based LEDs with ITO and graphene as TCSL, respectively, three critical factors of TCSLs should be discussed, namely, transmittance (T), sheet resistance ($R_{S}$) and contact resistance ($R_{C}$) between p-contact metal and p-GaN. The transmittance T of commercial ITO and of graphene are comparable in the spectral range from 400 nm to 800 nm. Below 400 nm, the transmission of ITO films experiences a sharp drop, whereas the graphene layers remain highly transparent (see Figure 1a).

Simulation with a finite element method reveals that reduced sheet resistance of the TCSL can enhance the current spreading length. Taking the sheet resistance of around 9 Ω/sq of a 400 nm thick ITO layer as one example, the current spreading length could be enhanced to ~164 µm compared to the value of ~116 µm in 200 nm ITO [53]. As summarized in Figure 1b, the thickness of ITO should exceed 100 nm to obtain a reasonable sheet resistance and thus a good device performance. In GaN-based LEDs the sheet resistance of ITO with a typical thickness t is 180 Ω/sq (t = 150 nm) [51], 30–48 Ω/sq (t = 240 nm) [12,54] and 20 Ω/sq (t = 280 nm) [35], respectively. On the contrary, multilayer graphene with a thickness of 10 nm has been theoretically predicted to have a sheet resistance of only 10 Ω/sq, which is two orders of magnitude lower than the experimental value (Figure 1b). In practical GaN-based LEDs, the sheet resistance of transferred SLG is not less than 660 Ω/sq [33,44,52] and for FLG not less than 220 Ω/sq [38,51,54,55,56]. Only by increasing the graphene layer number to >35, can the sheet resistance be reduced to around 100 Ω/sq [33]. However, due to the trade-off between sheet resistance and transparency, the optical transmittance of this multilayer graphene is below 20% for the whole UV-VIS spectral range and thus makes it inadequate for any TCSL application in visible or UV LEDs. The obstacle of achieving low sheet resistance in transferred graphene is attributed to the introduction of scattering centers for the charge carriers during the transfer process. These scattering centers include defects such as microcracks, wrinkles [57] and chemical residuals from PMMA [58].

As summarized in Table 1, the contact resistance of graphene/p-GaN is orders of magnitude higher than the typical ITO/p-GaN contact resistance of several mΩcm^2^ [18]. This difference in the contact resistance leads to 1–2 V higher forward voltages at typical input currents of 20 mA. By ultra-violet photoemission spectroscopy (UPS) measurements, the work function of the transferred few- or multi-layer graphene has been shown to range from 4.21 eV [31] to 4.5 eV [37], which is slightly lower than the reported work function value of 4.4–4.75 eV in 30–45 nm ITO films [61,62]. This slight difference in the work functions of graphene and ITO indicates that the main reason for the over 100-fold higher contact resistance between transferred graphene and p-GaN should not only originate from the work function mismatch but also from extrinsic factors due to the fabrication process. Different from evaporated ITO thin films on the substrate, graphene transferred onto the substrate suffers from bad adhesion [33] or microcracks, wrinkles and polymer residuals [58] that deteriorate the ideal contact formation between the transferred graphene and p-GaN. Details on the interface barrier between graphene and p-GaN will be discussed in Section 3.

Despite the apparent potential of graphene as a TCSL, device performance in GaN-based LEDs with transferred graphene as the TCSL is not yet convincing. This might be attributed to the unexpected high sheet resistance and the extremely high contact resistance at the graphene/p-GaN interface, both of which deviate widely from the theoretical value of several tens of Ω/sq and 10^−3^ Ωcm^2^, respectively. Both could be a result of the weakly controlled transfer process.

#### 2.1.2. Direct Growth of Graphene Films

Graphene directly grown on the p-side of GaN-based LEDs is expected to solve problems originating from the transfer process, such as bad adhesion, polymer residuals, defects and other damage introduced during transfer. Three main methods for direct integration of graphene layers as TCSL into GaN-based LED are reported: spray deposition of a graphene dispersion solution with a subsequent heating treatment (chemically converted graphene, CCG) [50,63], catalyzed graphene growth with W/Ni using rapid thermal annealing (RTA) post processing [64] and plasma enhanced chemical vapor deposition (PECVD) with [65,66] and without catalysts [67,68].

**Figure 2:**
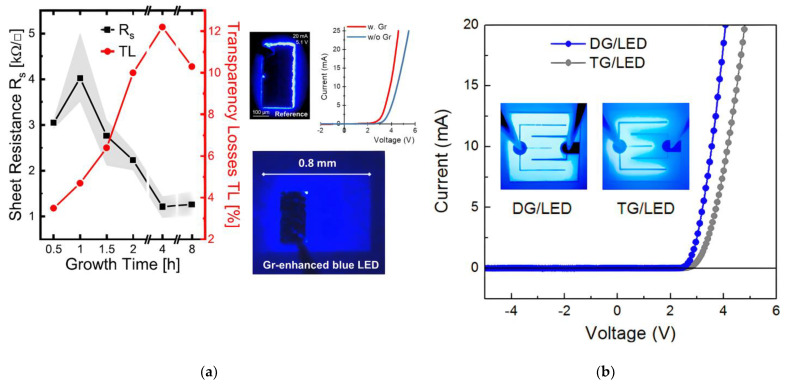
Directly grown graphene as TCSL integrated into GaN-based MQW LEDs: (**a**) The left panel shows the averaged value of the graphene sheet resistance (black squares) and the corresponding averaged transparency losses (red circles) as a function of growth time. The shaded areas represent the measurement errors. The right panel shows the current-voltage (I-V) characteristics and the light emission in a LED chip with graphene TCSL (red I-V curve and lower image) and without graphene TCSL (blue I-V curve and upper left image); (**b**) Optical images of light emission of a typical LED device (inset) and the I-V characteristics (plot) of a LED device with transferred graphene (i.e., TG, right inset, grey points in the I-V plot) and with directly grown graphene (i.e., DG, left inset, blue points in the plot). (**a**) Data from recent work and reproduced with permission from [68]. Copyright 2020 The Author(s). (**b**) Reproduced with permission from [67]. Copyright 2014 American Chemical Society.

As illustrated in Figure 2a, the transparency loss and sheet resistance of graphene layers can be tuned by altering the growth time during a PECVD process. With directly grown graphene as TCSL, a lower forward voltage and a better current spreading can be clearly observed compared to bare GaN-based LEDs (see Figure 2a, right). Figure 2b illustrates a lowered $V_{f}$ and an enhanced current spreading of a device with a PECVD grown graphene TCSL compared to a TCSL formed by transferred graphene in the same GaN-based LEDs [67]. It is argued that directly grown graphene shows a reduced contact resistance due to the in-situ contact formation to p-GaN.

Simulation results suggest that for a large current spreading length a TCSL with a low sheet resistance is essential [53]. However, due to the trade-off between $R_{S}$ and T, the sheet resistance of graphene for an acceptable transparency loss, e.g., <10%, is currently limited to ≥1 kΩ as shown in Figure 2a. Additionally, the contact resistance ($R_{C}$) between graphene and p-GaN of 0.15 Ωcm^2^ is still higher than the value of ITO/p-GaN; however, it is better than typical $R_{C}$ values of 0.22–5.5 Ωcm^2^ of transferred graphene on p-GaN [23,59,67]. More comparisons are summarized in Table 2.

Most directly grown graphene TCSLs in GaN-based LEDs are multilayer, with a strong variation in optical transmission. In case of CCG-grown graphene the optical transmission in the spectral range between 200 nm and 800 nm is higher than 85%, whereas the sheet resistance of above 2 kΩ/sq leads to a higher $V_{f}$ value of 6.4 V as compared to $V_{f,250\mathrm{nm}\;\mathrm{ITO}}$= 4.35 V of 250 nm ITO TCSL [63]. Similarly, graphene grown with a W/Ni catalyst shows an optical transmission above 84% in the UV-VIS range. However, its high sheet resistance of 6 kΩ/sq and the extraordinary high $V_{f}$ of 10 V at 0.4 mA need to be improved [64]. Only PECVD-grown graphene TCSLs with Co-catalyst [66] or without catalyst (see Figure 2a) show a $V_{f}$ below 5 V at 20 mA input current. In GaN-based UV LEDs [67], PECVD-grown MLG with a lower sheet resistance of 1.4 kΩ/sq and a relatively low contact resistance of 0.15 Ωcm^2^ as compared to transferred MLG leads to a reduced $V_{f}$ of 4.1 V, which is comparable or even better than for a 250 nm ITO TCSL.

However, the expected values of $R_{S}$ of ~10–100 Ω/sq and $R_{C}$ of ~mΩcm^2^ have not been achieved through either of the above-mentioned methods. The possible reasons are the relatively small grain size of graphene (~30 nm) and the high defect density, with distances between defects of around 2–5 nm [68], as reported for directly grown graphene. The grain boundaries and defects are all scattering centers and contribute to the increase of sheet and contact resistance. The reduction of these two kinds of scattering centers should be the main goal for directly grown graphene on the p-GaN LEDs, while keeping the growth temperature low.

### 2.2. Advanced Graphene-Based TCSLs

#### 2.2.1. Graphene Network with ITO

To achieve a better device performance, such as a lower $V_{f}$, and an enhanced LOP compared to ITO TCSL, graphene TCSL hybrid structures combined with different ITO configurations, namely ITO-nanodots (NDs), ITO thin films with 3–10 nm thickness, and 150 nm thick ITO films, in GaN-based LEDs are discussed in this section.

**Figure 3:**
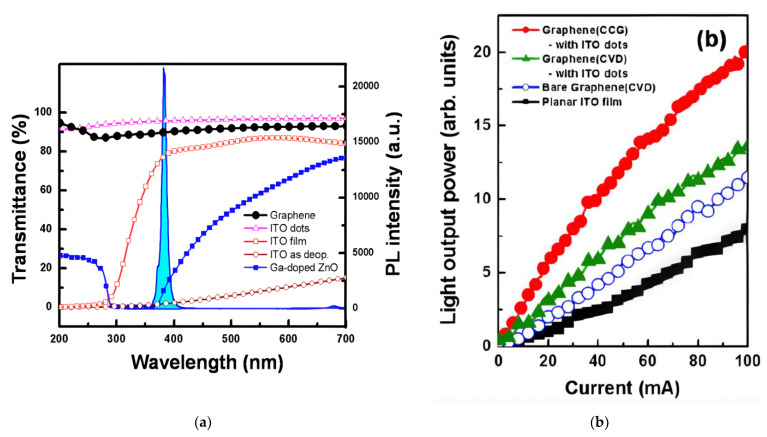
Graphene networked with ITO nanodots as TCSL integrated into GaN-based MQW LEDs: (**a**) the transmittance of a CCG graphene film (black full circles) compared to: ITO nanodots (pink triangles), annealed ITO film (red open squares), as-deposited ITO film (brown open circles), and Ga-doped ZnO (blue full squares); (**b**) the light output power in a typical LED device with four TCSLs: 250 nm thick ITO film (black squares), transferred CVD grown graphene (blue open circles), transferred CVD grown graphene networked with ITO nanodots (green triangles) and CCG graphene with ITO nanodots (red full circles). Reproduced with permission from [50]. Copyright 2011 Optical Society of America.

As previously mentioned, both transferred and directly grown graphene TCSL on the p-GaN LED substrate have a higher sheet resistance, and a higher contact resistance to p-GaN, than ITO. In contrast to ITO with, for example, 10% transparency at 300 nm, the transmission of graphene TCSLs remains sufficiently high (>80%) in the UV-VIS spectral range as illustrated in Figure 3a. However, the transmittance of ITO nanodots (NDs) with an average diameter of around 150 nm [63] has been proven to stay sufficiently high in a wide range of 200–700 nm (Figure 3a pink triangles). Additionally, the ITO-nanodots can provide texturing effects due to their spherical shape, which helps to reduce the total number of reflected photons, and thus increases the extraction efficiency [50,69].

As shown in Figure 3b, using this graphene/ITO nanodots hybrid structure as TCSL shows an over 150% enhancement of the LOP in comparison of ITO TCSL in GaN-based UV LEDs. Moreover, LOP of the directly grown graphene via CCG (Figure 3b, red circles) is higher than the value of transferred CVD graphene (Figure 3b, green triangles) with the same ITO nanodots. This improved LED performance can be observed in GaN-based blue LEDs as well, that is, a similar LOP value of a graphene hybrid TCSL as the one of ITO TCSL at an input current below 60 mA [63].

The 3–10 nm thin ITO films can have a high transparency of ≥94.5% from 320 nm to 780 nm, which is even better than 3LG as listed in Table 3. As summarized in Table 1 and Table 2, the contact resistance between ITO and p-GaN is much lower than that of graphene/p-GaN. Inserting the ITO film with a modified thickness between graphene and p-GaN could reduce the contact resistance in the graphene/ITO/p-GaN stack. The $V_{f}$ value is reduced to 3.90 V in GaN-based LEDs with a hybrid 3LG/7–10 nm ITO TCSL, which is much lower than the value of 6.76 V in LEDs with a 3LG-only TCSL [54].

Inserting several nm thick ITO films atop graphene as TCSL in blue LEDs could reduce the contact resistance at the p-side. This is indicated by the contact resistance of 3.72 × 10^−3^ Ωcm^2^ [70] compared to the typical value of around Ωcm^2^ in graphene-only TCSLs integrated in LEDs (see Table 1). Moreover, the LED performance with hybrid TCSLs is comparable or even better than with conventional ITO TCSLs in GaN-based blue LEDs. Coincidently, the hybrid TCSL consisting of the 150 nm thick ITO layer atop graphene shows a transmission T of around 94% at the emission wavelength of 475 nm. The sheet resistance of 41 Ω/sq of hybrid graphene/p-GaN is very comparable to the value of ITO/p-GaN, which results in only a 0.3 V difference of $V_{f}$ in LEDs with a hybrid TCSL than with an ITO TCSL. A GaN-based LED with this hybrid TCSL has a 145% LOP with respect to the one with ITO TCSL [71].

The hybrid structure of ITO/graphene or graphene/ITO as TCSL could thus increase the LOP through $R_{S}$ reduction and contact improvement while sustaining the transmission in different spectrum regions. Due to the low UV-absorption, the good contact to p-GaN and the texturing effect, graphene/ITO-nanodots could be a promising candidate as a TCSL in GaN-based UV LEDs.

#### 2.2.2. Graphene Network with Metallic Layers, Nanoparticles and Nanowires

**Figure 4:**
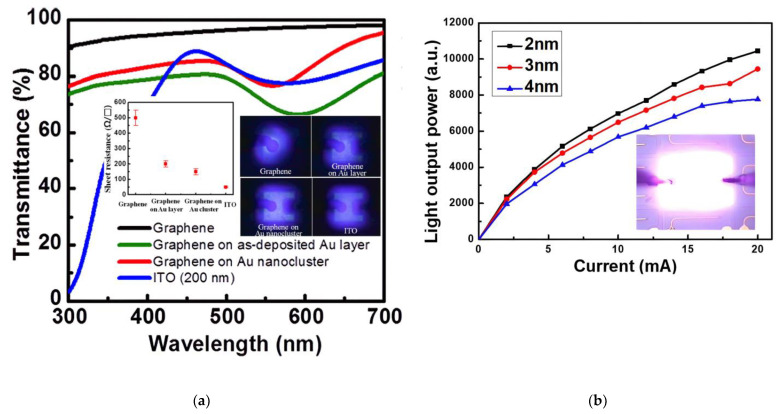
Graphene hybrid TCSL integrated into GaN-based UV MQW LEDs: (**a**) transmission of the graphene networked with Au layer or Au nanocluster compared with ITO and bare graphene. The left and right inset represent the corresponding sheet resistance and EL images of the with four different TCSLs; (**b**) LOP–current characteristics of a graphene/Ag nanoparticle hybrid as TCSL. The Ag nanoparticles (NPs) were formed by Ag films with a thickness varied from 2 nm to 4 nm. Inset: corresponding microscope image of an operating LEDs with graphene/Ag NP as TCSL (red circles in (**b**)) at 1 mA forward current. (**a**) Reproduced with permission from [12]. Copyright 2013 AIP Publishing LLC. (**b**) Reproduced with permission from [72]. Copyright 2018 IEEE.

A similar idea to enhance current spreading and light output power applies graphene hybrid structures combined with thin metal films, with nanoparticles or nanowires as a TCSL in GaN-based LEDs. This could preserve high transparency in a certain spectral range and could supply plenty of charge carriers, and therefore decreases the sheet resistance of hybrid structures. As shown in the right inset of Figure 4a, the sheet resistance of graphene hybrid TCSLs can be reduced from ~500 Ω/sq of the pristine graphene to ~200 Ω/sq by integrating a 2 nm thick Au film or down to ~150 Ω/sq by adding Au nanoclusters (NC). Both hybrid TCSLs have an additional 10–15% transparency loss compared to the graphene-only TCSL below 373 nm. Devices with the hybrid TCSLs exhibit a more uniform EL emission as compared to the pure graphene TCSL (see right inset of Figure 4a). Additionally, the contact characteristic is changed from non-linear (graphene/p-GaN) to ohmic (graphene/Au/p-GaN). The contact resistance of graphene/Au NC/p-GaN is 0.018 Ωcm^2^, more than four times lower than the value of graphene/Au layer/p-GaN [12]. As a result, both graphene/Au NC and graphene/Au layer hybrid TCSLs not only enhance the current spreading by reduction of the sheet resistance, but also improve the LOP and reduce $V_{f}$ by changing the p-contact properties while sustaining a high transparency.

For graphene hybrids, conductive materials, such as metal thin films, could either be deposited on the graphene surface or introduced as an interlayer between graphene and p-GaN. Both designs can have a transparency of around 75% between 350 and 700 nm [12,32,34] with metal layers thinner than 3 nm. The $R_{S}$ of the TCSLs can be reduced from 1250 Ω/sq (MLG) to 690 Ω/sq (3 nm Ni/MLG) [32] or from 500 Ω/sq (SLG) to 200 Ω/sq (SLG/2 nm Au). The corresponding contact resistance is summarized in Table 4: the insertion of a 2 nm Au thin film between MLG and p-GaN reduces $R_{C}$ from 1.3 to 0.24 Ωcm^2^ [31,73] or from 0.88 to 0.196 Ωcm^2^ [74]. Adding two metal layers, such as 1 nm Au / 1 nm Ni between SLG and p-GaN, $R_{C}$ decreases from 5.5 Ωcm^2^ to 0.6 Ωcm^2^ [34]. For metal thin films atop graphene, an optimized RTA step, e.g., around 500 °C in N_2_ or Ar atmosphere, can improve the contact characteristics. The reason might be that the RTA treatment eradicates the photoresist residuals [73], enhances the adhesion between layers [74] and lowers the Schottky barrier height (SBH) by formation of a metal-Ga solid solution at the interface [73]. This improvement could also be revealed by the reduced $V_{f}$ from 6.2 V (graphene-only TCSL) to 4.8 V (3 nm Ni/graphene hybrid TCSL) [32] and the enhanced EL intensity [34,75].

The graphene hybrid structure with metal nanoparticles (NPs) is normally formed by two steps. First, thin metal films were deposited between graphene and p-GaN or onto the graphene. Then the whole hybrid structure was exposed to a rapid thermal annealing step to form the nanoparticles. The thickness of the original metal films does not only define the dimension and density of nanoparticles [72] but also influences the performance of LEDs as shown in Figure 4b. The transmittance of this hybrid TCSL retains ≥82% [24]. Moreover, the sheet resistance of the TCSLs exhibits a reduction by adding the NP, e.g., from 500 Ω/sq (SLG) to 150 Ω/sq (SLG/Au NP) in GaN-based blue LEDs [12], or from 810 Ω/sq (SLG) to 280 Ω/sq (SLG/Ag NP) in GaN-based UV LEDs [52]. The contact resistance between the hybrid TCSL and p-GaN can be as low as 0.018 Ωcm^2^ [12] or even down to 10^−5^ Ωcm^2^ [72]. Additionally, the LOP and EL intensity are enhanced as well [24]. Some UV or near UV LEDs with SLG/Au NP [12] or SLG/Ag NP [52] as TCSL show higher LOP or similar EL intensity as compared to devices with a 200 nm ITO TCSL.

For the hybrid structure of graphene combined with nanowires (NWs), most studies integrate nanowires between graphene and p-GaN and sustain a high transmittance: 92.8% at 550 nm (FLG/Ag NW) [78], 86.3% at 375 nm (SLG/Ag NW) [79], as well as ≥88% from 400–800 nm (3LG/CNT) [56]. The sheet resistance of graphene TCSLs can be significantly reduced by inserting NWs. Take Ag NW as one example: $R_{S}$ can be as low as 30 Ω/sq in SLG/Ag NWs [79], while other works show a reduction of $R_{S}$ by a factor of two [56] or by 75% [78] compared to graphene-only TCSL. The specific contact resistivity of graphene TCSL/p-GaN can be reduced by more than 10 times to 0.105 Ωcm after adding Ag NWs [78]. As a result, the LED performance is significantly enhanced, with a 1–2 fold enhanced LOP with graphene/NWs compared to graphene-only TCSL [56,78] and a nearly four-fold improvement of the EL peak intensity [12].

In summary, graphene hybrid structures with metal thin films, nanowires or nanoparticles can reduce the sheet resistance and improve the contact property of TCSLs in GaN-based LEDs. However, the transmittance of the hybrid structure is kept above 80% only in nanowires and nanoparticles, whereas it is around 70% for a graphene/metal thin film hybrid as TCSL. This limits the improvement of LED performance.

#### 2.2.3. 0D or 3D Graphene Configuration

Different from the 2D graphene layer, two new types of graphene nanostructures, namely zero-dimensional (0D) graphene quantum nanodots (GQDs) and three-dimensional (3D) graphene foam, were also applied to enhance the GaN-based LED performance.

**Figure 5:**
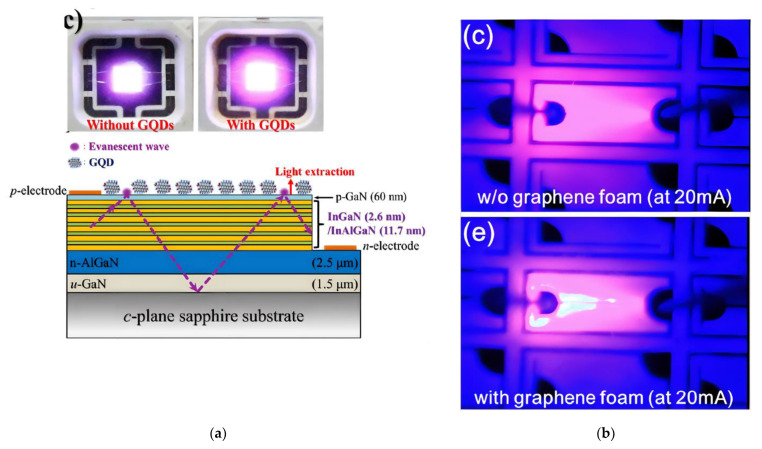
0D GQD as top layer and 3D graphene foam as TCSL integrated into GaN-based MQW LEDs: (**a**) The upper part is the optical image of an operating LED device without (left) and with 0D graphene GQD (right). The lower part illustrates a schematic cross-sectional view. The violet arrow denotes the direction of the light propagation; (**b**) EL images in a LED device without (upper) and with (lower) graphene foam. (**a**) Reproduced with permission from [45]. Copyright 2017 The Author(s), (**b**) Reproduced with permission from [46]. Copyright 2013 AIP Publishing LLC.

The implementation of QNDs in a GaN-based LED device structure is illustrated schematically in Figure 5a. GQDs can exhibit enhanced UV absorption compared to 2D graphene due to the plasmonic effect [45]. The lateral dimension of GQDs is less than 100 nm [80], which enables collective electron movement named as surface plasmons [81,82]. Due to this collective movement of charge carriers in GQDs, the transverse part of the totally reflected UV light at the interface between air and p- or n-type doped GaN can be absorbed by GQDs. During this process, holes or electrons may be generated in the graphene and injected back into the active layers to contribute to the recombination process. As a consequence, an enhanced EL intensity, an improved LOP, and an increase of the EQE from 6.9% (without GQDs) to 11.8% (with GQD) at 100 mA was obtained [45]. Applying nitrogen doped GQDs on the n-GaN side for vertical GaN-based LEDs, both LOP and EQE are enhanced by a factor of three compared to vertical LEDs without GQDs [60].

A 3D foam structure of graphene, which has hierarchical macro- and meso-porous structures, was considered to be able to sustain a high electrical conductivity and a good mechanical stability [83], as well as a low mass density and a large surface area [84,85]. One approach produces the 3D foam graphene on a 3D Cu foam by CVD with a subsequent transfer process onto a GaN-based blue LED substrate [46]. Compared with the reference LED (see upper panel of Figure 5b), the current spreading region is enhanced by integrating the 3D graphene foam (see Figure 5b, lower panel). Moreover, the LOP is enhanced by 14% and the $V_{f}$ is reduced from 6.61 V to 4.85 V at 100 mA. However, the transmittance of the 3D graphene foam is 71 % at 438 nm, with a further drop below 400 nm. The sheet resistance is 800 Ω/sq on quartz [46]. Due to the restricted optical and electrical properties, 3D graphene foam as a TCSL does not show a significant improvement in the performance compared to other hybrid structures listed above.

### 2.3. Current Spreading Effect

**Figure 6:**
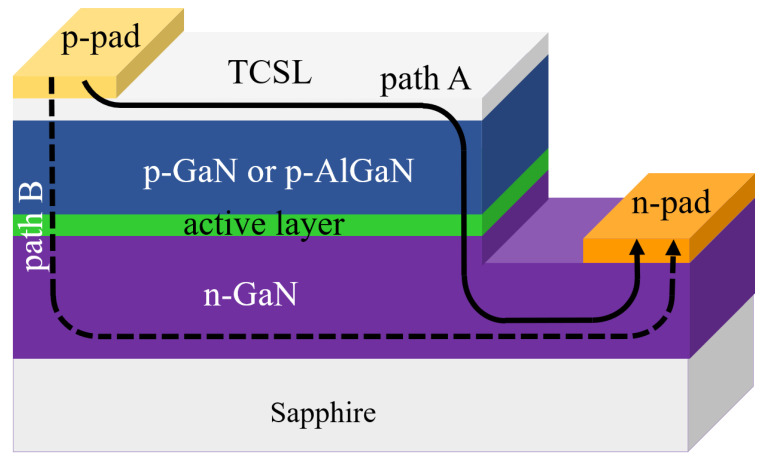
Current flow through path A (solid line) and path B (dashed line) in a typical GaN-based LED.

To describe the current spreading effect by inserting a TCSL, a current spreading length ($L_{S}$) is defined, over which the current density decreases to 1/e of its initial value [86]. The larger $L_{S}$, the better the current spreading performance. The current flow from the p- to the n- electrode in a LED mesa structure has two choices as illustrated in Figure 6: if the lateral current injection is dominating, i.e., the current flows mainly along the path A, current spreading happens. If the current flows mainly through path B, current crowding occurs around the p-contact pad. The ratio of the current of the two paths can be defined as a factor Q, i.e., $Q=I_{B}/I_{A}$. Therefore, Q<1 means that the current spreading at the p-side dominates. For a given Q value, the $L_{S}$ can be expressed as [53]:

(1) $$L_{s}=\sqrt{\frac{2n_{ideal}k_{B}T_{op}}{J_{0}e*\left(\frac{\rho_{TCSL}}{t_{TCSL}}+Q*\frac{\rho_{n-GaN}}{t_{n-GaN}}\right)}}\;,$$

where $n_{ideal}$ is the diode ideality factor, which is normally in the range of 1.05–1.35 [87]. The sheet resistance of the TCSL and of the n-GaN layer can be given as $\rho_{TCSL}$/$t_{TCSL}$ and $\rho_{n-GaN}$/$t_{n-GaN}$, respectively, where t and ρ are the thickness and the specific resistivity of the TCSL and the n-GaN layer, respectively. $J_{0}$ is the total injection current, e is the elementary charge, $k_{B}$ is the Boltzmann constant and $T_{op}$ is the operation temperature. Based on this equation, the sheet resistance of a TCSL has an impact on $L_{S}$: if the sheet resistance decreases, $L_{S}$ increases. Attempts to reduce the sheet resistance of TCSL should enhance the current spreading effect.

As outlined in Section 2.2.1 and Section 2.2.2, graphene hybrid TCSLs exhibit a reduced sheet resistance due to the insertion of highly conductive materials. The total resistance of this hybrid TCSL is thus lower than graphene-only TCSL [88]. Metallic interfacial layers and nanowires can form extra conduction paths within the hybrid structure [89]. As the simulation suggests, the conductivity is increased and the sheet resistance of the whole TCSL is reduced, which leads to an enlarged current spreading length [53]. Due to the current flow across the p-GaN, the contact resistance on the p-GaN side can also impact the current spreading and the LED performance.

## 3. Contact Engineering between Graphene and GaN

### 3.1. Motivation for Contact Engineering between Graphene/p-GaN

A Schottky barrier is expected to form when graphene is in direct contact with p-GaN because of the work function mismatch. Meanwhile, graphene can be doped, leading to a change in its work function and thus of the Schottky barrier height (SBH) at the graphene/p-GaN interface. XPS results reveal an SBH at the graphene/p-GaN interface of 2.06 eV [90], which indicates the p-type doping of graphene.

Some other factors can influence the SBH values as well, for example, the layer number of graphene. This has been shown for graphene deposited on n-GaN. As revealed by XPS, the binding energy position of the Ga 3d core level is 21.5 eV (bare GaN), 21.4 eV (SLG coated), 21.3 eV (2LG coated) and 21.1 eV (MLG coated). This core level shift impacted by the number of graphene layers is related to the Fermi level shift of graphene and thus reveals a change of the SBH at the graphene/n-GaN interface, i.e., the SBH of SLG/n-GaN is smaller than in case of MLG/n-GaN [91]. In contrast, the experimental results show that the contact resistance $R_{C}$ of SLG/p-GaN (~10^−1^ Ωcm^2^) is higher than the value of MLG/p-GaN (5.8 × 10^−2^ Ωcm^2^) [33]. Another example is that a short time thermal annealing could lead to an interdiffusion of ions, such as Ga or Cr, into the graphene/GaN interface and may form a new and higher metal/p-GaN barrier [92]. Therefore, control of the SBH and its stability and robustness are of pivotal importance. Two practical methods are in use for SBH control at the graphene/p-GaN interface in GaN-based LEDs. One is the p-doping of graphene (Section 3.2), which increases the work function of graphene and thus can bring down the SBH. Another approach is integrating a thin oxide film into the graphene/p-GaN interface (Section 3.3). By keeping the tunnel barrier thin enough, a decrease of the contact resistance on the p-side can be obtained.

### 3.2. Chemical Doping of Graphene

The intentional p-type doping of graphene TCSLs applied in GaN-based LEDs can be divided into the noble-metal ion doping (Section 3.2.1) and non-metal acid doping (Section 3.2.2).

#### 3.2.1. Metal Doping

**Figure 7:**
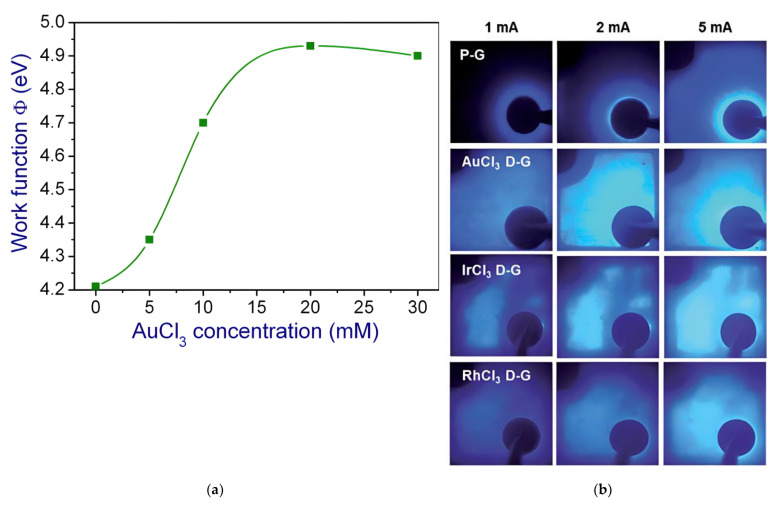
Graphene doped with metal-chlorides integrated as TCSL into GaN-based MQW LEDs: (**a**) work function of doped graphene films as function of AuCl_3_ concentration; (**b**) light emission images in a typical LED device with different dopants in graphene, namely AuCl_3_, IrCl_3_ and RhCl_3_ compared to pristine graphene (P-G) from top to bottom, and at various current levels, namely 1 mA, 2 mA and 5 mA from left to right. (**a**) Reproduced with permission from [73]. Copyright 2012 IOP Publishing Ltd. (**b**) Reproduced with permission from [38]. Copyright 2014 The Royal Society of Chemistry.

Use of various metal-chlorides, such as AuCl_3_ [31,73,93,94], IrCl_3_ and RhCl_3_ [38], was attempted for doping graphene. All these dopants show a clear enhancement of the graphene work function. As illustrated in Figure 7a the doping concentration has a significant impact on the graphene work function. A non-linear increase of the work function from 4.2 eV to around 4.93 eV is observed as the AuCl_3_ concentration increases from 0 to 20 mM (1 mM = 10^−3^ mol/L). However, a further increase of the AuCl_3_ concentration to 30 mM will slightly reduce the graphene work function [73]. Doping with Au-, Ir- and Rh-chlorides with the same concentration of 20 mM exhibit a similar increase of the graphene work function to 4.9, 4.95 and 5.1 eV, respectively [38]. The current spreading effect at different input currents is illustrated in Figure 7b. An enlarged light emitting area is conspicuous for graphene TCSL doped by all the three dopants. For a brief overview of the doping effect on graphene, properties such as T, $R_{S}$ and work function (Φ) are listed in Table 5, together with their impact on the LED performance.

As revealed by XPS, the noble-metal-chloride doping process is a charge transfer process, where Au^3+^ is reduced to Au^0^ through electron depleting in graphene [95,96]. Au^0^ atoms tend to form nanoparticles during this process, and the nanoparticle size increases as the dopant concentration increases [95]. However, these nanoparticles also serve as scattering centers for electromagnetic waves, which could lead to a reduction of the transmission. For example, the transmittance of undoped 3LG at 363 nm is 87.9%, while after doping this value decreases to 86.4% (5 mM), 85.1% (10 mM) and 82.9% (20 mM), respectively [93].

After doping, the sheet resistance of graphene layers can be reduced due to the added charge carriers in the graphene. Doping concentration and type of dopants can impact the final sheet resistance. With the same doping species, the initial value of $R_{S}$ of undoped 3LG of 466.1 Ω/sq is lowered to one-third while doping by 5 mM AuCl_3_, and further down to 158.5 Ω/sq (10 mM) and 112.4 Ω/sq (20 mM), respectively [93]. For the same doping concentration of 20 mM, metal-chlorides (MCl_3_) with different metal ions show variations in the sheet resistance: The sheet resistance of undoped 4L graphene $R_{S,\;4\mathrm{LG}}$ of 220 Ω/sq can be reduced to 110 Ω/sq (AuCl_3_), 105 Ω/sq (IrCl_3_), and 140 Ω/sq (RhCl_3_), respectively, by doping.

Generally, the high contact resistance of around Ωcm^2^ originates from the SBH at the graphene/p-GaN interface and leads to a higher forward voltage. After AuCl_3_ doping, the work function of 12L graphene rises from $\Phi_{12\mathrm{LG}}$ = 4.21–4.24 eV (undoped graphene) to $\Phi_{p-12\mathrm{LG}}$ = 4.93 eV (p-type doped graphene). Thus, the contact resistance of 12LG/p-GaN decreases from 1.3 Ωcm^2^ (undoped) to 0.4 Ωcm^2^ (p-type doped graphene), and the forward voltage from 6.59 V (undoped) to 5.55 V (p-type doped graphene) [31,73]. This trend can be seen not only in blue LEDs, but also in UV LEDs. GaN-based UV LEDs with undoped graphene TCSL show a contact resistance of $R_{C}$ = 1.3 Ωcm^2^ and a forward voltage of $V_{f}$ = 5.85 V. After AuCl_3_ doping, $R_{C}$ and $V_{f}$ are as low as 0.24 Ωcm^2^ and 3.98 V, respectively. This is comparable to $V_{f,200\;\mathrm{nm}\;\mathrm{ITO}}$ = 3.92 V in a reference device with a 200 nm thick ITO as TCSL. However, the graphene-enhanced device shows a 120% LOP compared to the value of a device with an ITO TCSL [94]. The current injection is also dependent on the AuCl_3_ doping level: at an injection voltage of 8 V, the injection current is increased by 48% (5 mM), 63% (10 mM) and 73% (20 mM), respectively in a GaN-based UV LED [93].

#### 3.2.2. Non-Metal Doping

**Figure 8:**
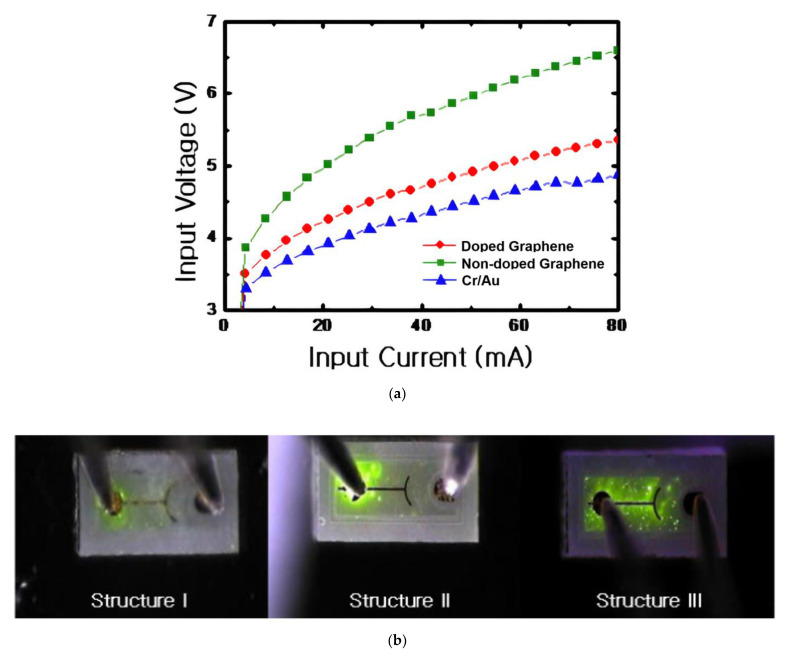
Graphene doped by HNO_3_ acid as TCSL integrated into GaN-based LEDs: (**a**) I-V characteristic and (**b**) light emission images at an injection current of 5 mA with three different p-contacts: Cr/Au without TCSL (blue triangles and structure I), Cr/Au/graphene (green squares and structure II) and Cr/Au/doped graphene (red dots and structure III). Reproduced with permission from [98]. Copyright 2013 IOP Publishing Ltd.

One study with HNO_3_ acid doping of graphene shows an enhancement of the current spreading in GaN-based LEDs as well [98]. A significantly reduced forward voltage in GaN-based LEDs with graphene TCSL at a given current is obtained after doping as shown in Figure 8a. Figure 8b illustrates a clearly enhanced current spreading in LEDs especially with doped graphene TCSL (structure III). Moreover, inserting undoped MLG TCSL improves LOP by 42.3% compared to the bare LEDs, and 30.8% extra enhancement can be obtained after HNO_3_ doping of MLG.

Due to some missing data in [98], such as sheet resistance, contact resistance and optical transmission before and after the non-metal doping, no direct explanation can be derived for the reason for the performance enhancement. In other works, the influence of HNO_3_ doping in graphene layers has been illustrated. Pristine SLG fabricated by the roll-to-roll method shows over 91% transmittance from 200 nm to 1000 nm [25]. After HNO_3_ doping, the maximal transmittance loss of graphene is only 5%, observed at 200 nm, while a high transmission of over 92% is sustained in a range of 300–1000 nm. This is highly promising for applying HNO_3_ doped graphene as TCSL in GaN-based UV-VIS LEDs as compared to metal chloride doped graphene, as in the latter the formed metal nanoparticles have generally a UV absorption (see Section 3.3). Similar to the case of metal chloride doping, the sheet resistance can be reduced by doping with HNO_3_ as well: e.g., from 272 Ω/sq (pristine SLG) to 108 Ω/sq (HNO_3_ doped SLG) or from 40 Ω/sq (pristine 4LG) to 30 Ω/sq (HNO_3_ doped 4LG) [25]. As measured by UPS, FLG doped with HNO_3_ shows an increased work function from 4.52 eV to 5.31 eV as the HNO_3_ concentration is raised from 0 to 100 wt% [99]. This increase of the work function could lower the SBH and decrease the contact resistance of the p-type doped graphene/p-GaN, leading to an improvement of LOP after HNO_3_ doping of transferred MLG.

### 3.3. Inserting Thin Dielectric Films at the Graphene/p-GaN Interface

**Figure 9:**
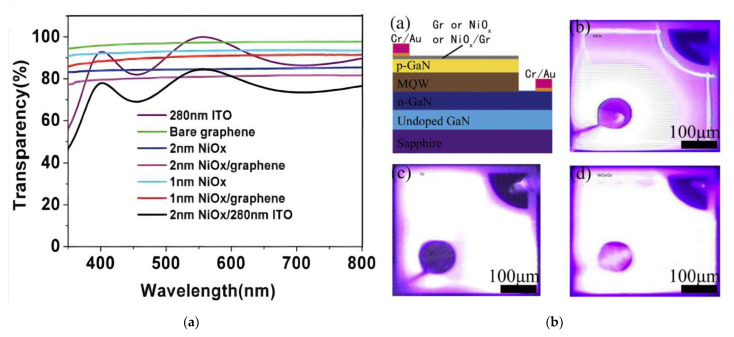
Graphene with NiO_x_ interlayer as TCSL in GaN-based MQW LEDs: (**a**) transmission spectra of different layer combinations: NiO_x_ (1 nm and 2 nm), NiO_x_/graphene, graphene, NiO_x_/ITO, and 280 nm ITO; (**b**) cross-sectional image (upper left) and EL images of LEDs with different TCSLs: NiO_x_ (upper right), graphene (lower left), and NiO_x_/graphene (lower right). (**a**) Reproduced with permission from [35] Copyright 2012 The Royal Society of Chemistry. (**b**) Reproduced with permission from [15]. Copyright 2015 Elsevier Ltd.

Another effective method for modifying the transport barrier between graphene and p-GaN is introducing a thin metal oxide film with a high work function. NiO_x_, as a p-type metal oxide with a work function higher than 5 eV, is a most promising and well-investigated candidate for this application [15,33,35,56,74,100]. However, since the metal oxide mostly has a high sheet resistance and also reduces the transmission in the UV-VIS range, the thickness of this oxide should be well controlled. As shown in Figure 9a, the transparency of 1 nm and 2 nm NiO_x_ thin films can be >90% and >83% in the spectral range of 350–800 nm, respectively. Its current spreading performance is illustrated in Figure 9b, where pure graphene TCSLs, pure NiO_x_ TCSLs and hybrid graphene/NiO_x_ TCSLs are compared. NiO_x_ is normally produced by oxidation of Ni thin films via annealing in air [15,35] or O_2_ containing ambient [74,100]. As suggested by XPS measurements NiO_x_ consists of NiO and Ni_2_O_3_ phases [35].

By reducing the layer thickness of Ni, the transparency of the graphene/NiO_x_ stack can be increased. Taking FLG with T = 95% in the visible region as one example, the transmission of graphene/NiO_x_ can be increased from around 80% to nearly 90% by reducing the Ni thickness from 2 nm to 1 nm [35]. A post-metallization annealing was reported to improve the adhesion between graphene and p-GaN and thus enhance the contact characteristics [74]. After transferring graphene onto NiO_x_/p-GaN, some works [33,74,100] thus applied a post-graphene-transfer annealing aimed at forming a low resistance contact to p-GaN. This, however, deteriorates the transmission of graphene/NiO_x_.

Various performance data for graphene/NiO_x_ integrated in GaN-based LEDs are listed in Table 6. After transferring FLG onto NiO_x_/p-GaN LEDs, a thermal annealing in N_2_ for 300 s brings the transmission from over 95% at 450 nm down to around 93.6% (RTA at 350 °C), 88.6% (RTA at 450 °C) and 87% (RTA at 550 °C), respectively [100]. This decrease could be explained by the valence change of Ni^2+^ or Ni^3+^ to Ni^0^, which indicates a metal phase formation in NiO_x_. Coincidently, the contact resistivity of the FLG/NiO_x_/p-GaN trilayer stack changes from 8.2 × 10^−2^ Ωcm initially to 5.6 × 10^−2^ Ωcm after RTA at 550 °C) [100]. Therefore, the trade-off between transparency loss and enhancement of the contact property should be taken into consideration, especially for graphene/NiO_x_/p-GaN with insertion of NiO_x_. Other works without RTA prove a direct reduction of the contact resistance at the p-side after introducing NiO_x_ [15,35]. The bilayer stack FLG/NiO_x_ (1 nm or 2 nm) preserves the same sheet resistance value as the bare graphene, whereas the contact resistance decreases dramatically from 10^−2^−10^−1^ Ωcm^2^ in FLG/p-GaN to 5.9 × 10^−4^ Ωcm^2^ in FLG/NiO_x_/p-GaN. The roughly two orders of magnitude reduction in the contact resistance is attributed to changes in the band bending in p-GaN and a decrease of the contact barrier width due to the NiO_x_ [35].

Most works show an improvement of EL or LOP through NiO_x_ insertion compared with the reference sample with bare graphene. However, for GaN-based blue LEDs, the LOP is still not as good as with ITO TCSL. Even though $R_{C}$ of FLG/NiO_x_/p-GaN is as low as 5.9 × 10^−4^ Ωcm^2^, the lowered T of around 80% and the sheet resistance of 1.2 kΩ make the LOP of FLG/NiO_x_/p-GaN lower than in the case of a TCSL of a 280 nm thick ITO [35]. Therefore, attaining a superior performance for blue LEDs with graphene-based TCSL requires a further reduced $R_{S}$ and a sustained transmittance.

## 4. Advanced Device Engineering

**Figure 10:**
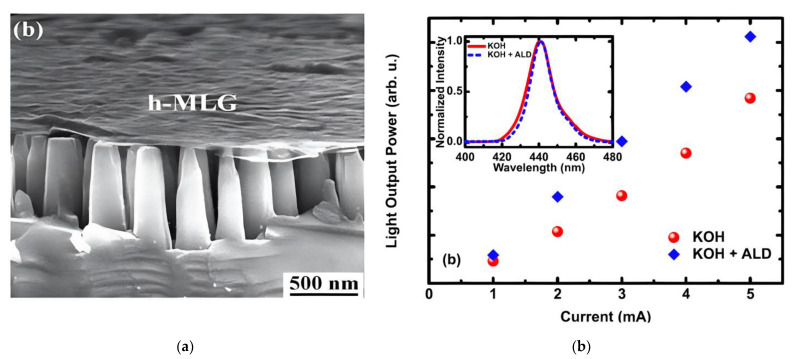
Graphene as TCSL in nanopillar MQW LEDs. (**a**) Scanning electron microscopy image of the nanopillars with highly homogeneous multilayer graphene; (**b**) light output power as a function of input current level with graphene (red circles) and graphene/Al_2_O_3_ (blue diamonds). The inset shows the respective EL spectra. (**a**) Reproduced with permission from [102]. Copyright 2011 The Royal Society of Chemistry. (**b**) Reproduced with permission from [105]. Copyright 2016 IOP Publishing Ltd.

Graphene TCSLs are also applied for GaN-based devices with complex 3D architecture, such as vertical light emitting nanorods, nanopillars and photonic crystal structures with air holes. Due to its mechanical robustness and stretchability [55,67], graphene works well for current spreading and connecting different light emitting parts in such structures. Nanopillar and nanorod devices have large light emitting areas, and can release strain in the MQW and enhance light extraction due to increased scattering and reduced reflection at the sidewalls [101,102,103]. One application of multi-stacked four-layer graphene that encases nanopillars is illustrated in Figure 10a. This approach avoids polymer filling between pillars or in air-holes [104]. Passivation by the atomic layer deposition (ALD) of, e.g., Al_2_O_3_ on graphene could further increase the LOP of GaN-based nanopillar LEDs by 30% as shown in Figure 10b.

Transmittance and sheet resistance of transferred graphene in these advanced devices are similar to, or even better, than in the conventional planar device structures listed in Table 7. For example, $R_{S}$ of 3LG on a planar SiO_2_ substrate is 1000 Ω/sq but only 300 Ω/sq on the nanorods [101]. A similar reduction of $R_{S}$ in 2LG from 300 Ω/sq to 107 Ω/sq is obtained by changing the substrate from SiO_2_ to a triangle lattice air hole photonic crystal (PC) GaN-based LEDs [104]. A direct experimental explanation for this $R_{S}$ reduction is that the suspended few-layer graphene possesses a higher sheet conductance (~2500 $e^{2}/h$) compared to the supported graphene (~20 $e^{2}/h$) [106]. This high value of quantized conductance in suspended graphene could be attributed to the ballistic transport [107]. The smaller value of $R_{S}$ leads to a homogeneous electroluminescence with current spreading effect in nanorods [101] and in photonic crystal structures [104].

Applying 3LG as TCSL, the GaN-based nanorod or nanopillar devices show 32% improvement of the LOP compared to planar structures and a higher LOP than the same nanorod structure with Ni/Au as TCSL [101]. The GaN-based airhole photonic crystal with 2LG as TCSL shows 60% higher LOP in comparison to the same structure without graphene [104]. Performing the ICP etching process during device fabrication before transferring the graphene layer can avoid the ICP damage on graphene [38] and thus increase the LOP by 55.3% [103] (see Table 7).

**Figure 11:**
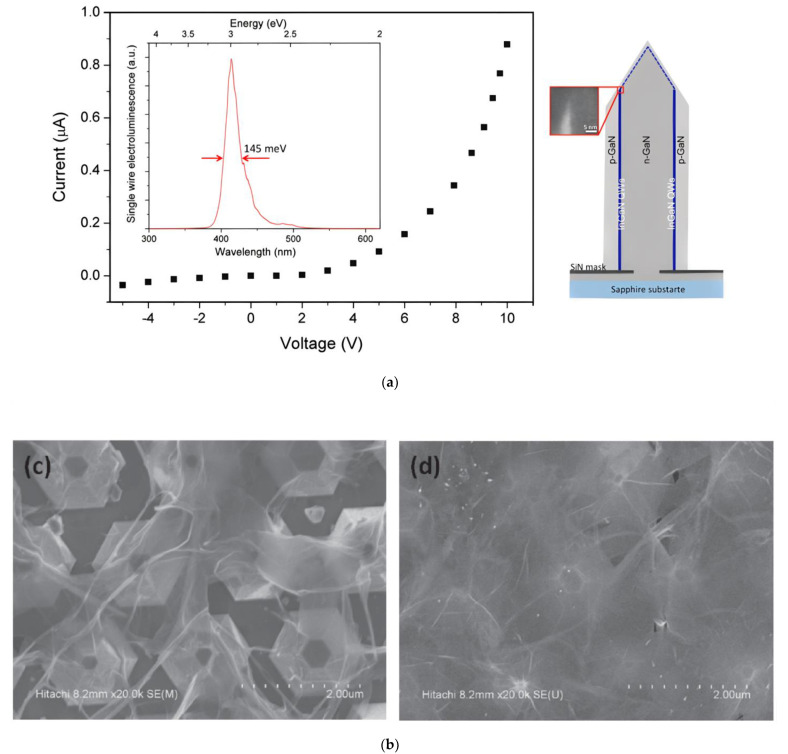
Graphene as TCSL in core-shell nanowire MQW LEDs: (**a**) EL (inset) and I-V characteristic (main plot) in a single nanowire LED with graphene (left) and schematic image of the nanowire structure (right); (**b**) SEM image of graphene transferred 3 (left) and 4 (right) times onto a large-area pyramid core-shell nanowire LED. (**a**) Reproduced with permission from [109]. Copyright 2014 American Chemical Society. (**b**) Reproduced with permission from [108]. Copyright 2013 The Japan Society of Applied Physics.

Different from the above architectures, horizontal core-shell GaN-based LEDs places n-GaN in the center of each pillar or pyramid, which is then surrounded by the MQW and p-GaN. This geometry can also provide strain relaxation, realize light emission in one unit and promise a reduction of the spectral broadening due to a well-organized growth of the nanowire size [108,109]. Single graphene can be transferred either once onto a single nanowire LED as shown in Figure 11a, or multiple times onto a multiwire LED array as visualized in Figure 11b. The multiple transfer process is necessary for a complete coverage on the multiwire LED arrays. A negligible spectral shift of the EL and a homogeneous EL emission can be observed [108].

Monolithic GaN-LED arrays also require a highly transparent conductive layer as interconnection for the micro-LEDs to reduce the optical loss during light emission. Using graphene to connect p-GaN and n-GaN of two neighboring micro-LEDs, the LOP is 7.4% higher than in the same device structure with a Cr/Pt/Au metal interconnect [110]. As a transparent conductive electrode, graphene can improve the light extraction in GaN-based core-shell LEDs and monolithic GaN-based LED arrays.

## 5. Summary

Graphene with its unique properties is thought to be a promising material as a transparent current spreading layer in GaN-based LEDs for solving the current crowding issue due to the intrinsically low conductivity in the p-cladding layer. In the early development stage, sheet resistance and contact resistance of as-grown graphene were several orders of magnitude larger than conventional ITO, whereas the transparency of both materials in the visible spectral region was similar. The high sheet resistance in graphene films was attributed to the introduction of damage during the fabrication process, whereas the high contact resistance could originate from the intrinsic large work function difference between graphene and p-GaN, and bad adhesion and graphene damage by the transfer process.

Graphene was modified in various ways in the past decade, aiming at improving the performance of GaN-based LEDs with graphene TCSL. To avoid defects, wrinkles and chemical residuals introduced from the transfer process, direct growth methods were developed. A reduction of the sheet resistance can be realized through hybrid structures, that network graphene with conductive participants in the form of layers, nanoparticles or nanowires. However, this enhancement typically results in a reduced transparency, which could weaken the LED performance. Improvements in the contact resistance at the graphene/p-GaN interface have been achieved through work function engineering of graphene through p-doping and through adding an ultrathin oxide layer such as NiO_x_. In case of metal doping, however, the formation of agglomerated metal nanoparticles could reduce light transmission. When inserting an ultrathin oxide layer such as NiO_x_ between graphene and p-GaN, both stoichiometry and thickness of NiO_x_ have to be well controlled to avoid a high transmission loss. All attempts to decrease the sheet resistance of graphene and the contact resistance on the p-side could deteriorate the transparency. Graphene TCSLs has been shown to be attractive even for new emerging devices with 3D architecture. However, systematic studies are still vacant, and a rational design strategy for the further development of graphene as TCSL in GaN-based LEDs is required.

## Figures and Tables

**Table 1:** Usage of transferred single layer (SLG), few-layer (FLG) and multilayer (MLG) graphene TCSLs in GaN-based LEDs. Transmittance values of TCSLs are measured at (@) a certain wavelength or in a spectral range, and the electrical parameters are measured at (@) a certain input current.

TCSL	Process	$R_{S}\;\left(Ω/\mathrm{sq}\right)$	$R_{C}\;\left(Ω{\mathrm{cm}}^{2}\right)$	***T*** (%)	EL (nm)	$V_{f}(V)\;\;@\;20\;\mathrm{mA}$	Performance Comparison
SLG	CVD on Cu [49]	-	-	~95 @ 460 nm	460	5.87	$V_{f,250\mathrm{nm}\;\mathrm{ITO}}\;$= 3.4V @ 20 mA: LOP_SLG_ = 125%LOP_250nm ITO_
	CVD on Cu [33]	994–1400	≈10^−1^	≥95 @ 350–800 nm	450	>5	LOP _SLG_ < LOP_5nmNi/5nm Au_ @ 0–350 mA
	CVD on Cu [52]	810	-	>90 @ 300–800 nm	380–400	5.9	$V_{f,200\mathrm{nm}\;\mathrm{ITO}}\;$= 3.92 V
	CVD on Cu [15]	-	260 Ω	~92 @ 400–700 nm	blue-purple	6.4	@ 20 mA: EL_SLG_ ≈ 57%EL_200 nm ITO_
	CVD on Cu [44]	658	-	97 @ 380 nm	380	5.2 ^HD^5.7 ^LD^	@ 20 mA: EL_SLG/HD_ ≈ 164%EL_SLG/LD_
FLG	Scotch-tape [22]	-	-	-	368	26.5 @ 1 mA	SQW vertical LEDs
	CVD on Cu [23]	280–850 [51,55]	-	≥80 @ 300–800 nm	372	10 @ 5 mA	SQW@ 10 V: I_FLG_ > I_w/o FLG_
	vertical cold wall CVD on Cu [54] 3LG	300–350	-	≥81.9 @ 320–780 nm	blue	6.76	$V_{f,240\mathrm{nm}\;\mathrm{ITO}}$= 3.60 V
	CVD on Cu [38] 4LG	220	-	87 ^b^, 85 ^a^ @ 470 nm	-	5.9 @ 10 mA	ICP damage
	CVD on CNT/Cu [56]	1122	-	95 @ 400–800 nm	452	6.93	LOP_FLG_ = 0.52 mW @ 4 mA
	CVD on Cu [50]	290	-	~78 (ITO)~90 (2LG) @ 380 nm	380	5.9	$V_{f,250\mathrm{nm}\;\mathrm{ITO}}\;$= 4.35 V LOP_2LG_ = 140%LOP_ITO_ @ 100 mA
MLG	CVD on Ni [51]	620	-	85 @ 400–800 nm	443	5.6	$V_{f,150\mathrm{nm}\;\mathrm{ITO}}\;$= 3.8 V LOP_MLG_ = 63%LOP_ITO_
	CVD on Cu [59]	-	0.220.59 ^1^	96 @ 550 nm	495	≥5.38	$\mathrm{RTA}\;\mathrm{impact}\;\mathrm{on}\;V_{f}$
	CVD on Ni [33]	108–80	0.058	15 @ 450 nm	450	3.1	>35LG
3D foam	CVD on 3D Cu [46]	800	-	71 @ 438 nm	438	4.85 @ 100 mA	@ 100 mA: EL_3Dfoam_ = 114%EL_w/o 3Dfoam_ LOP_3Dfoam_ > LOP_w/o 3Dfoam_
0D graphene quantum dots (GQD)	Pulsed laser ablation [45]	-	-	-	387	-	LOP_GQD_ > LOP_w/o GQD_ EQE_GQD_ = 11.8% EQE_w/o GQD_ = 6.9% @100 mA
	Hydrothermal method [60]	-	-	-	450	2.58	LOP_GQD_ = 125%LOP_w/o GQD_ EQE_GQD_ = 7.5% @15 mA

^1^ RTA. ^a^ after or ^b^ before inductively coupled plasma (ICP) etching. ^HD^ heavily or ^LD^ lightly doped p-GaN layer is applied.

**Table 2:** Usage of directly grown graphene (Gr) TCSLs in GaN-based LEDs.

TCSL	Process	$R_{S}\;\left(Ω/\mathrm{sq}\right)$	$R_{C}\;\left(Ω{\mathrm{cm}}^{2}\right)$	***T*** (%)	EL (nm)	$V_{f}\;(V)\;\;@\;20\;\mathrm{mA}$	Performance Comparison
Gr	CCG @ 240 °C, MLG [63]	2200	-	≥85 @ 200–800 nm	460	6.4	$V_{f,250\mathrm{nm}\;\mathrm{ITO}}$~3.64 V
	CCG @ 240 °C, MLG [50]	2200	-	≥85 @ 200–700 nm	380	-	$V_{f,250\mathrm{nm}\;\mathrm{ITO}\;}$= 4.35 V
	PECVD @ 600 °C, 5–6 L [67]	1400	0.15	≥65 @ 350–800 nm	365	4.1	LOP= 7.46 mW @ 20 mA
	Annealing of a-C @ 700 °C MLG [64]	6000	-	84.5 @ 260 nm 91.1 @ 550 nm	blue	10 @ 0.4 mA	W/Ni catalyst
	PECVD on Co @ 600 °C [65]	-	-	>65 @ 400–1000 nm	-	5.2	$V_{f,w/o\;\mathrm{Gr}}\;$= 7.7 V
	PECVD ≤800 °C ^1^ [68]	1020–1300	-	>88 @ 350–750 nm	blue	4.4 ^1^	$V_{f,\frac{w}{o}\mathrm{Gr}}$= 5.1 V
	PECVD on Co @ 700 °C [66]	-	-	>85 @ 400–1000 nm	-	4.7	$V_{f,w/o\;\mathrm{Gr}}\;$= 7.6 V

^1^ Data are from the recent work in this group.

**Table 3:** Usage of hybrid graphene TCSLs combined with various ITO configurations: nanodot (ND), thin and thick films.

TCSL	Process	$R_{S}\;\left(Ω/\mathrm{sq}\right)$	$R_{C}\;\left(Ω{\mathrm{cm}}^{2}\right)$	***T*** (%)	EL (nm)	$V_{f}(V)\;\;@\;20\;\mathrm{mA}$	Performance Comparison
Gr ^d^/ ITO ND	CCG [63]	2200	-	≥85 (Gr) >90 (ITO ND) @ 200–800 nm	460	3.66	@ ≤ 60 mA: LOP_Gr/ITO ND_ ≈ LOP_ITO_ @ > 60 mA:LOP_Gr/ITO ND_ < LOP_ITO_
	CCG [50]	2200	-	≥85 (Gr) >90 (ITO ND) @ 200–800 nm	380	4.42	@ 100 mA: LOP_Gr/ITO ND_ = 250%LOP_250 nmITO_
2LG/ ITO ND	CVD on Cu [50]	290	-	95 @ 400–800 nm	380	4.9	@ 100 mA: LOP_2LG/ITO ND_ = 170%LOP_250 nmITO_
3LG/ 7–10 nm ITO	vertical cold wall CVD on Cu [54]	300–350	-	≥94.5 (7–10 nm ITO) ≥81.9 (3LG) ≥78.6 (3LG/10 nm ITO) @ 320–780 nm	blue	3.90	$V_{f,240\mathrm{nm}\;\mathrm{ITO}}$= 3.60 V
3.3 nm ITO/SLG	CVD on Cu [70]	-	3.72 × 10^−3^ (ITO/SLG/p-GaN, ^1^) 1.52 × 10^−2^ (ITO/p-GaN, ^1^)	-	453	3.053.14 (ITO)	@ 20 mA: LOP_3_._3TO/SLG_ = 21.2 mW LOP_ITO_ = 19 mW EQE_3_._3TO/SLG_ = 39.1% EQE_ITO_ = 35.3%
150 nm ITO/SLG	CVD on Cu [71]	739 ^S^ 41	0.31 (ITO/SLG/p-GaN) 0.22 (ITO/p-GaN)	>94 ^S^ @ 300–700 nm ~94 (ITO, ITO/SLG) @ 475 nm	475	3.6 3.3 (ITO)	@ 100 mA: LOP_150nmITO/SLG_ = 145%LOP_150nm ITO_

^1^ RTA at 500 °C in N_2_ for 5 min. ^d^ denotes the direct growth as distinguished from transfer. ^S^ SLG.

**Table 4:** Graphene combined with various metal configurations: layer, nanoparticle (NP), nanocluster (NC), nanodot (ND), nanowire (NW), nanorod (NR). Stack sequence from left to right is from upmost to p-GaN. CNT denotes carbon nanotube.

Hybrid with	Process	$R_{S}\;\left(Ω/\mathrm{sq}\right)$	$R_{C}\;\left(Ω{\mathrm{cm}}^{2}\right)$	***T*** (%)	EL (nm)	$V_{f}\;(V)\;@\;20\;\mathrm{mA}$	Performance Comparison
layer	CVD on Ni [31,73] MLG/2 nm Au	1150	1.3(MLG/p-GaN) 0.24 (MLG/Au/ p-GaN)	averaged 72 (MLG) @ 350–800 nm	455	4.63 4.26 ^1^	$V_{f,240\mathrm{nm}\;\mathrm{ITO}}=4\;V$ $V_{f,MLG}$ = 6.59 V
	PECVD on Cu [57] SLG/Ni|Au (1 nm|1 nm)		-	≥ 90 ^S^ @ 200–800 nm	blue	3.03 (Gr/Ni|Au)	@ 20 mA: $V_{f,200\mathrm{nm}\;\mathrm{ITO}\;}$= 3.01 V LOP_Gr/Ni|Au_ = 9.36 mW LOP_ITO_ = 10.15 mW
	CVD on Cu [76] 2LG/Ni|Au (1 nm|1 nm)	760 ^S^ 380 (2LG)	-	-	-	3.4 (2LG/Ni|Au)	$V_{f,240\mathrm{nm}\;\mathrm{ITO}\;}$= 3.6 V
	CVD on Cu [34] SLG/Au/Ni (1 nm/1 nm)	100–200	5.5(SLG/p-GaN) ^1^ 0.6 (SLG/Au/Ni/ p-GaN) ^1^	≥95 ^S^ ≥78 (SLG/Au/Ni) @ 350–800 nm	446	3 (Gr/Au/Ni)	@ 3 V: EL_SLG/Au/Ni_ = 500% EL_SLG or bare_
	CVD on Cu or Ni [74] SLG/2 nm Ni SLG/2 nm Au	-	0.88(SLG/p-GaN)^1^ 0.196 (SLG/Au/ p-GaN)^1^	≥85 (Ni) ≥80 (Au) ≥97.5 ^S^ @ 400–800 nm	blue	4.5 ^S^	-
	CVD on Cu [32] 3 nm Ni/Gr	1250 (Gr) 690 (Ni/Gr) ^1^	-	> 90 (Gr) @ 300–700 nm 75 (Ni/Gr) 86 (ITO) @ 460 nm	460	6.2 (Gr) 4.8 (3Ni/Gr)	$V_{f,\;150\;\mathrm{nm}\;\mathrm{ITO}}$= 3.5 V @ 20 nmA: EL_3Ni/Gr_ ≈ EL_Gr_ < EL_ITO_
	CVD on Cu [32] 3 nm Ni/Gr	1250 (Gr) 690 (Ni/Gr) ^1^	-	74 (Ni/Gr) 70 (150 nm ITO) @ 380 nm	380	13.2 (Gr) 7.1 (3Ni/Gr)	$V_{f,\;\;150\;\mathrm{nm}\;\mathrm{ITO}}\;$= 5 V @ 5 mA: EL_Ni/Gr_ = 83% EL_ITO_
	CVD on Cu [12] SLG/2 nm Au	500 ^S^ 200(SLG/Au)	0.08 (SLG/Au/ p-GaN)	92.8 ^S^ 78 (SLG/Au) @ 373 nm	373	7.2 ^S^ 4.25(SLG/Au)	$V_{f,\;200\;\mathrm{nm}\;\mathrm{ITO}\;}$=3.94 @ 20 mA: EL_SLG/Au_< EL_ITO_
	CVD on Cu [75] AZO/2 nm Ni/SLG	70 (AZO)	$R_{C}$(AZO/Ni/SLG/p-GaN)=150% *R_C_*(AZO/Ni/p-GaN)	65 (AZO/2Ni/SLG) 66 (AZO/2Ni) @ 386 nm	386	AZO/Ni: 4.6 ^S^ 5.8 (w/o Gr)	$\Phi_{SLG}\;$= 4.85 eV $\Phi_{Ni}\;$= 4.48 eV @50 mA: EL_AZO/Ni/SLG_ = 195% EL_AZO/Ni_
NP, NC, ND	CVD on Ni [24] Ag NP/MLG	-	-	89 (MLG) 82(MLG/Ag * NP) @ 460 nm	460	4.5 (MLG) 3.4 (MLG/ Ag * NP)	@ 100 mA: LOP_Ag NP/MLG_ = 270%LOP_MLG_
	CVD on Cu [12] SLG/Au NC	500 ^S^ 150(SLG/Au)	0.018 (SLG/Au/ p-GaN)	92.8 ^S^ 82.7 (SLG/Au) @ 373 nm	373	7.2 ^S^ 4.02 (SLG/Au NC)	$V_{f,\;200\;\mathrm{nm}\;\mathrm{ITO}}$= 3.94 @ 20 mA: EL_SLG/Au_ = 110% EL_ITO_
NP, NC, ND	CVD on Cu [52] SLG/Ag NC	810 ^S^ 280 (SLG/Ag)	-	>95 ^S^ @ 300–800 nm 84 (SLG/Ag) @ 380 nm	380–400	5.9 ^S^ 4.06 (SLG/Ag)	$V_{f,\;200\;\mathrm{nm}\;\mathrm{ITO}}$= 3.92 V @ 20 mA: EL_Gr/Ag_ ≈ EL_ITO_
	CVD on Cu [72] MLG/Ag NDs	551 (MLG)	0.25 (TCSL/p-GaN)^2Ag^ 2.1×10^−5^ (TCSL/p-GaN)^4Ag^	82.5 (MLG/ND)^2Ag^ 69.5 (MLG/ND)^4Ag^ @ 365 nm	365	7.4 (MLG/ND)^2Ag^ 5.9 (MLG/ND)^4Ag^	$V_{f,\;\mathrm{ITO}\;}$=4.2 V @ 20mA: LOP_MLG/2Ag_ = 131%LOP_MLG/4Ag_
NW, NR	CVD on Cu [77] ZnO NW/SLG	100–200	5 ZnO/SLG/p-GaN	97 ^S^ 90 (ZnO NW/SLG) @ 450–800 nm	446	2.7(ZnO/SLG) 2.9 (ZnO) @ 7.5 mA	@ 2.8 V: LOP_ZnO/SLG_ = 166%LOP_SLG-LED_
	CVD on Cu [78] 2–3LG/Ag NW	1350 (FLG) 367 (FLG/Ag NW)	1.06 Ωcm (FLG/p-GaN) 0.105 Ωcm (FLG/Ag/p-GaN)	96.2 (Ag NW) 92.8 (FLG/Ag NW) @ 550 nm	blue	11.8 (FLG) 6.6 (FLG/Ag)	@ 20 mA: LOP_2–3LG/Ag_ = 295% LOP_2–3LG_
	CVD on Cu [79] SLG/Ag NW	500 ^S^ 50 (Ag NW) 30 (SLG/Ag)	-	93 ^S^ 90.2 (Ag NW) 86.3 (SLG/Ag NW) @ 375 nm	375–378	10.9 ^S^ 6.7 (Ag NW) 4.48 (SLG/Ag)	@ 20 mA: EL_SLG/Ag NW_ = 589% EL_SLG_
	CVD on CNT/Cu [56] 3LG-CNT	1122 (3LG) 533 (3LG-CNT)	-	95 (3LG) 88 (3LG-CNT) @ 400–800 nm	452	6.12 (3LG-CNT) 6.93 (3LG)	@ 4 mA LOP_3LG_ = 0.52 mW LOP_3LG-CNT_ = 1.3 mW

^1^ RTA. ^S^ SLG. * Patterned micro-circle. ^4Ag^ or ^2Ag^ denotes ND formed by a 2 nm or 4 nm thin Ag film.

**Table 5:** Usage of p-type doped graphene (p-Gr) as TCSL in GaN-based LEDs. The doping concentration is given in mM.

TCSL	Process	$R_{S}\;\left(Ω/\mathrm{sq}\right)$	$R_{C}\;\left(Ω{\mathrm{cm}}^{2}\right)$	***T*** (%)	EL (nm)	$V_{f}\;(V)\;@\;20\;\mathrm{mA}$	Performance Comparison
SLG/ Ga:ZnO (GZO)	CVD [97] AuCl_3_ doping	170 (20–30 mM)	-	≥ 80 (2.5–30 mM) @ 425 nm	425	-	@ 10 mA: EL_p-SLG/GZO_ ≥ 220%EL_GZO_
BLG	CVD on Cu [94] AuCl_3_ doping	-	0.24 (p-BLG/p-GaN) 1.3(BLG/p-GaN)	92 (BLG) 88.5(10 mM) @ 380 nm	380	3.98 (10 mM) 5.85 (BLG)	$V_{f,200\mathrm{nm}\;\mathrm{ITO}}$= 3.92 V @ 100 mA: EL_p-BLG_ = 120%EL_200 nm ITO_ $\Phi_{p-\mathrm{BLG}}\;$= 4.90 eV $\Phi_{\mathrm{BLG}\;}$= 4.51 eV
FLG	CVD on Cu [93] 3LGA uCl_3_ doping	466.1 (3LG) 175.5 (5 mM ^1^) 158.5 (10 mM ^1^) 112.4 (20 mM ^1^)	-	87.9 (3LG) 86.4–85.1–82.9 (5–10–20 mM) @ 363 nm	363	@ 6.5 V $I_{3\mathrm{LG}}$ ≈ 0.65 mA $I_{p-3\mathrm{LG}}$ ≈ 1.5–3 mA	EL_p-3LG_ ≥ EL_3LG_
	CVD on Cu [38] 4LG AuCl_3_ doping	780 ^S^ 220 (4LG) 105–140 (20 mM)	-	96 ^S,b^ 87 (4LG) ^b^ 83–84 (20 mM) ^b^ @ 470 nm	-	-	$\Phi_{p-4\mathrm{LG}\;}$= 4.9–5.1 eV $\Phi_{4\mathrm{LG}}\;$= 4.2 eV
MLG	CVD on Ni [36] 9LG AuCl_3_ doping	1000 (9LG) 203 (5 mM) 103 (20 mM)	-	89 (9LG) 85 (5 mM) 78 (20 mM) @ 400 nm	400	4.73 (9LG) 3.94 (5 mM) 3.86 (20 mM) @ 0.4 mA	@ 0.5 mA: EL_p-9LG_ = 193.8%EL_9LG_ $\Phi_{p-9\mathrm{LG}}\;$= 4.77–5.12 eV $\Phi_{9\mathrm{LG}}\;$= 4.42 eV
	CVD on Ni [31,73] >12LGA uCl_3_ doping	1150 (MLG) 476 (20 mM)	1.3 (MLG/ p-GaN) 0.4 (doped MLG/p-GaN 0.24(MLG/Au/p-GaN)	72 ^A^ (MLG) ~67.5 (20 mM) @ VIS	blue	6.59 (MLG) 5.55 (p-MLG) 4.63(MLG/Au) 4.26(MLG/Au ^1^) 3.96 (p-MLG/Au ^1^)	$V_{f,240\mathrm{nm}\;\mathrm{ITO}\;}$= 4 V $\Phi_{\mathrm{Au}}\;$= 5.1 eV $\Phi_{12\mathrm{LG}}\;$= 4.21–4.24 eV $\Phi_{p-12\mathrm{LG}}\;$= 4.93 eV
	CVD on Ni [98] ≈10LG HNO_3_ doping	-	-	-		~3.9 (w/o TCSL) ~5 (10LG) ~4.25 (p-10LG)	$\Phi_{10\mathrm{LG}\;}$= 4.21 eV $\Phi_{p-10\mathrm{LG}}\;$= 4.93 eV LOP_w/o Gr_ = 8.67 mW LOP_10LG_ = 12.34 mW LOP_p-10LG_ = 17.83 mW @ 80 mA

^1^ RTA. ^A^ Averaged value. ^b^ before ICP. ^S^ SLG. w/o TCSL denotes without TCSL.

**Table 6:** Usage of graphene combined with NiO_x_ as TCSL in GaN-based LEDs.

TCSL	Process	$R_{S}\;\left(Ω/\mathrm{sq}\right)$	$R_{C}\;\left(Ω{\mathrm{cm}}^{2}\right)$	***T*** (%)	EL (nm)	$V_{f}(V)\;@\;20\;\mathrm{mA}$	Performance Comparison
SLG/ NiO_x_	CVD on Cu [15] NiO_x_: 3 nm Ni RTA in air	-	260 Ω (SLG/p-GaN) 2000 Ω (NiO_x_/p-GaN) 75 Ω (SLG/NiO_x_/p-GaN)	>91 ^S^ >89 (NiO_x_) >81(SLG/NiO_x_) @ 400–700 nm	blue	6.4 (SLG) 4.5 (SLG/NiO_x_) 3.9 (ITO)	$\Phi_{p-\mathrm{GaN}}$ = 5.5–5.9 eV @ 20 mA: EL_SLG/NiOx_ = 85% EL_200nm ITO_ = 150% EL_SLG_
	CVD on Cu or Ni [74] 2 nm NiO_x_: Ni RTA in O_2_ ambient	-	8.8×10^−1^ (Gr/p-GaN, ^1^)	≥97.5 ^S^ ≥95.5(SLG/NiO_x_) ≥90.5 (SLG/NiO_x_,^1^) @ 400–800 nm	blue	4.5 (Gr) 3.16 (Gr/NiO_x_) 3.6 (NiO_x_)	$\Phi_{\mathrm{Gr}/\mathrm{NiOx}}$≈ 5.53 e V@ 20 mA: LOP _Gr/NiOx_^1^ ≈ 70%LOP_Gr_
FLG/ NiO_x_	CVD on Cu [35] 2–3L G1–2 nm NiO_x_: Ni RTA in air	>10 kΩ (1–2 nm NiO_x_) ~1.2 kΩ (FLG, FLG/1 nm NiO_x_, FLG/2 nm NiO_x_)	5.9× 10^−4^ (Gr/NiO_x_/p-GaN) 10^−2^–10^−1^ (Gr/p-GaN)10^−3^ (ITO/(NiO_x_)/p-GaN)	90–83 (1–2 nm NiO_x_) >95 (FLG) >85(FLG/1NiO_x_) >78(FLG/2NiO_x_) @ 350–800 nm	blue	6.15 (FLG) 3.65 (FLG/NiO_x_) 3.2 (ITO)	@ 20 mA: LOP_280 nm ITO_ ≥ LOP_FLG/NiOx_ = 157%LOP_FLG_
	CVD [100] 3LG NiO_x_: 2 nm Ni RTA, N_2_:O_2_ = 4:1	-	(5.3–7.6)× 10^−4^ (Gr/NiO_x_/p-GaN, ^1^ 550 °C)	>95 (FLG/NiO_x_) @ 400–800 nm FLG/NiO_x_,^1:^ 93.6 (350 °C) 88.6 (450 °C) 87 (550 °C) @ 450 nm	450	5.4 (FLG) FLG/NiO_x_,^1^: 4.4 (350 °C) 3.9 (450 °C) 3.5 (550 °C)	$\Phi_{\mathrm{FLG}}\;$= 4.56 eV $\Phi_{\mathrm{FLG}/\mathrm{NiOx}}\;$= 4.98 eV $\Phi_{\mathrm{FLG}/\mathrm{NiOx},450\;{}^{\circ}C}$ = 4.67 eV @ 100 mA: LOP_FLG/NiOx,350 °C_ > LOP_Ni/Au_ > LOP_FLG/NiOx, 550 °C_
FLG- CNT/ NiO_x_	CVD on CNT/Cu [56] 3LG2 nm NiO_x_	1122 (3LG) 1110(3LG/CNT) * 533 (3LG-CNT) **	-	95 (3LG) 95 (2NiO_x_) 88 (3LG/CNT) ** @ 400–800 nm	452	6.93 (3LG) 6.12 (3LG-CNT) 5.12 (3LG-CNT/NiO_x_)	$\Phi_{2\mathrm{nm}\;\mathrm{NiOx}}\;$≈ 5.1 eV @ 4 mA: LOP_3LG_ = 0.52 mW LOP_3LG-CNT_ = 1.30 mW LOP_3LG-CNT/NiOx_ = 2.08 mW
MLG/ NiO_x_	CVD on Ni [33] >35LG 2 nm NiO_x_	994–1400 ^S^ 108–80 (MLG) 1338 (MLG^2^) 37 (Ni) ≈300 (3LG)	3.7× 10^−4^ (Ni/Au/p-GaN) 5.8× 10^−2^ (MLG/p-GaN) 3.7× 10^−1^ (MLG ^2^/p-GaN) 2.1× 10^−2^ (MLG/ NiO_x_/p-GaN) ≤10^−1^ (SLG/p-GaN)	≥97.5 ^S^ ≥98 (2NiO_x_) 89 (3LG) 87 (3LG/NiO_x_) 71 (Ni/Au) 15 (MLG) @ 450 nm	450	≈3.1 (MLG/NiO_x_) 3.2 (3LG/NiO_x_) ≈3.1 (MLG)2.9 (Ni/Au) >5 (SLG) 3.37–3.47( SLG/NiO_x_)	@ 100 mA: LOP_3LG/NiOx_ > LOP_Ni/Au_ > LOP_SLG_ > LOP_MLG/NiOx_

^1^ RTA after graphene transfer. ^2^ O_2_ plasma thinned MLG. * Stacked layers. ** Composed layers. ^S^ SLG.

**Table 7:** Usage of graphene as TCSL used in advanced GaN-based LEDs.

Device	Process	$R_{S}\;\left(Ω/\mathrm{sq}\right)$	$R_{C}\;\left(Ω{\mathrm{cm}}^{2}\right)$	***T*** (%)	EL (nm)	$V_{f}\;(V)\;@\;20\;\mathrm{mA}$	Performance Comparison
1D nano-pillar	CVD on Cu MLG, RTA^1^ [102]	550 ^S^ 215 (MLG)	-	96 ^S^ 87 (MLG)@ 450 nm	451	4.6	mechanically robust no EL shift
nanorod (NR)	CVD on Cu MLG transfer before (I) and after (II) ICP, RTA^1^ in N_2_, 200 °C, 15 min [103]	-	-	-	456	11.5 (w/o Gr) 8.9 (I) 7.0 (II)	@ 20 mA: EL_II_ = 155.3%EL_I_
	Rapid CVD on Cu FLG [101]	3000 ^S^ 1000 (3LG)[54] 300 (3LG) on ND	0.8–1.8 (Au/Ti/3LG/p-GaN)	81.9 (3LG) @ 320 nm [54]	blue	5.2 (ND) 6.7 (flat mesa)	WPE_3LG-ND_ = 5.2% > WPE_Ni/Au-ND_ @ 20 mA: LOP_3LG-ND_ = 132%LOP_3LG-flat mesa_
	CVD on Cu MLG [105]	-	-	-	405	5.5 (KOH and KOH/ALD) @ 1 mA	PL_KOH+ALD_ = 130%PL_KOH_ = 180%PL_as etched_ EL_KOH+ALD_ = 130%EL_KOH_
Air hole photonic crystal (PC)	CVD 2LG [104]	~600 ^S^ ~300 (2LG) on planar SiO_2_ 107 (2LG) on PC	-	nearly 95 (2LG) 85 (Ni/Au/2LG) @ 460 nm	-	planar LEDs: 3.3 (w. 2LG) 4.3 (w/o Gr) PC LEDs: 3.4 (w. 2LG) 4 ^S^ 4.9 (w/o Gr)	@ 20 mA: LOP_2LG PC_ = 6.2 mW LOP_2LG,planar_ =4.5 mW LOP _PC_ = 3.8 mW
Core-shell	CVD on Cu MLG RTA^1^ [108]	-	-	≥9 5@ 425–800 nm	478	8.8	Current spreading improved
	CVD on Cu SLG [109]	-	-	-	494 ^2^ 415 ^3^	4 (turn-on)	EL appears at 6 V, 0.16 µA

^1^ RTA after graphene transfer. ^2^ Low current injection. ^3^ High current injection. ^S^ SLG.

## Data Availability

No new data were created or analyzed in this study.
